# Non-invasive brain stimulation for patient with autism: a systematic review and meta-analysis

**DOI:** 10.3389/fpsyt.2023.1147327

**Published:** 2023-06-29

**Authors:** Annan Liu, Chao Gong, Bobo Wang, Jiaxing Sun, Zhimei Jiang

**Affiliations:** ^1^Jiamusi University Affiliated No.3 Hospital, Jiamusi, China; ^2^Jiamusi Medical College, Jiamusi, Heilongjiang, China; ^3^Jiamusi University College of Rehabilitation Medicine, Jiamusi, Heilongjiang, China

**Keywords:** autism, non-invasive neurostimulation, transcranial direct current stimulation, transcranial magnetic stimulation, meta-analysis

## Abstract

**Objective:**

To comprehensively evaluate the efficacy of non-invasive brain stimulation (NIBS) in patients with autism spectrum disorder (ASD) in randomized controlled trials (RCT), providing a reference for future research on the same topic.

**Methods:**

Five databases were searched (Pubmed, Web of Science, Medline, Embase, and Cochrane library) and tracked relevant references, Meta-analysis was performed using RevMan 5.3 software.

**Results:**

Twenty-two references (829 participants) were included. The results of the meta-analysis showed that NIBS had positive effects on repetitive and stereotypical behaviors, cognitive function, and executive function in autistic patients. Most of the included studies had a moderate to high risk of bias, Mainly because of the lack of blinding of subjects and assessors to treatment assignment, as well as the lack of continuous observation of treatment effects.

**Conclusion:**

Available evidence supports an improvement in some aspects of NIBS in patients with ASD. However, due to the quality of the original studies and significant publication bias, this evidence must be treated with caution. Further large multicenter randomized double-blind controlled trials and appropriate follow-up observations are needed to further evaluate the specific efficacy of NIBS in patients with ASD.

## 1. Introduction

Autism spectrum disorder (ASD) is a neurodevelopmental disorder that is typically characterized by social communication and interaction impairments, restricted and repetitive behavior or interests, often accompanied by a range of psychiatric problems such as attention deficit hyperactivity disorder and sleep disorders. Clinically usually presents with cognitive, behavioral, emotional, and expressive language impairments (1–3). In recent years, the incidence of the disease is increasing, the global prevalence rate is about 7.6‰ (4). According to 2020, Recent data from the Centers for Disease Control and Prevention show that The overall ASD prevalence aged 8 years rises to 1 in 44, which is already more than the sum of the world’s three major diseases (AIDS, cancer, diabetes) (5). ASD starts in early childhood and the relevant symptoms can last for a lifetime, causing a heavy emotional, financial, and medical burden on patients, their families, and society, which has become a serious public health issue (6–8). At present, the origin of ASD is not clear, and it is usually caused by multiple factors alone or together, among which genetic and environmental factors play an important role in its occurrence and development (9).

Regarding the pathology of ASD, structural and connectivity differences have been identified in the brains of ASD patients. Scholars in imaging have proposed many theories of abnormal brain connectivity in ASD patients to try to explain the pathological mechanisms of the brains of ASD and their abnormal social behaviors. There are three examples, Cohen et al. proposed the amygdala theory of autism, the evidence of this theory shows that there is a significant correlation between the amygdala and social behavior. Abnormalities of the amygdala (including damage, volume abnormalities, microscopic lesions, etc.) can lead to socio-intellectual deficits, and social dysfunction of ASD is closely related to it (10). Rubenstein et al. proposed the hypothesis of abnormal brain activation or inhibition of ASD, This hypothesis believes that the key nervous system excitation/inhibition ratio increases under genetic factors or environmental factors, resulting in cortical “noise” is the key to the occurrence of autism, thus providing a new way to treat ASD by inhibiting neural excitability (11). In recent years, Just et al. proposed the hypothesis, of disconnection of the cerebral cortex in ASD, that is the functional connections of various cerebral regions in the cortex were weakened, which revealed that the brain information coordination and integration ability of ASD was weakened (12). In particular, high cell counts in the prefrontal cortex, enlarged amygdala volume, and reduced functional connectivity between the anterior cingulate cortex (ACC) and the dorsolateral prefrontal cortex (DLPFC) are considered to be specific neurostructural features in children with ASD (13).

In order to change and improve the developmental trajectory of ASD, numerous scholars have conducted extensive research and discussion on various aspects of autism rehabilitation treatment. Most of these studies are based on behavioral interventions accompanied by related medications, but the results are often unsatisfactory (14). Therefore, it is necessary to explore a new therapeutic approach to complement the behavioral intervention-based treatment model in order to improve the intervention effect. NIBS is a kind of emerging therapy, and that is increasingly being used in adult and pediatric neurological rehabilitation (15). NIBS interventions refer to non-invasive and painless transcranial stimulation neuromodulation techniques, including transcranial magnetic stimulation (TMS) and transcranial direct current stimulation (TDCS) (16). TMS works by converting coil currents into intermittent, localized pulsed magnetic fields that act on the cerebral cortex, causing local depolarization and firing of neurons. TMS mainly includes single-pulse TMS (sTMS), paired TMS (pTMS), and repetitive TMS (rTMS). Theta-Burst Stimulation (TBS) also known as theta-burst transcranial magnetic stimulation, a variation of TMS, consists of a 50-Hz triplet pulse burst with a 200-ms interburst interval typically at 80% of the Active Motor Threshold (AMT) (17). iTBS, a type of TBS (18), is similar to the Long-term Potentiation (LTP) of rTMS, which achieves excitability by high-frequency stimulation (> 1 Hz), and could induce excitability in the primary motor cortex (19). Among them, the use of rTMS in the treatment of pediatric and adolescent diseases is more applied, which refers to the repeated stimulation of specific areas of the scalp with the same pattern and time intervals to achieve the treatment effect (20–22). rTMS includes high-frequency stimulation (≥5 Hz) and low-frequency stimulation≤ (1 Hz), high-frequency rTMS produces long-duration enhancement, which increases cortical excitability, and low-frequency rTMS produces long-duration inhibition, which decreases cortical excitability (23). In recent years, rTMS has been tried as an adjunctive therapy for ASD and can improve some of the core symptoms of ASD (24).

tDCS can cause hyperpolarization of resting membrane potential and regulate the activity of neural networks by using direct electrical currents to stimulate a targeted cortical area, to achieve the therapeutic purpose (25). Compared with TMS, tDCS does not directly induce brain activity but changes the excitability of spontaneous brain activity by subliminal regulation of neuronal membrane potential (26). In general, the anode of tDCS increases neuronal excitability and the cathode decreases it. Continuous regulation of neural flexibility induced by tDCS may be the basis for its treatment of psychiatric disorders (27). At present, there are many studies and applications of tDCS on speech impairment, social impairment, stereotyped behavior, and emotional changes in ASD patients of different ages (28, 29). tDCS provides appropriate stimulation to the patient based on the accurate positioning given by the pathological analysis of ASDs to achieve relief of symptoms.

Clinical studies have shown that the NIBS techniques for the treatment of patients with ASD were effective. Luckhardt et al. (30) systematically searched for the database before 2020, and six eligible randomized, sham-controlled clinical trials of tDCS in patients of ASD were included. The analysis indicated that tDCS improved significantly ASD patients’ cognitive and social-communication skills. Recently, 10 studies were rated as low risk of bias in a systematic review by Zhang et al. (31). Four investigated the efficacy of tDCS on ASD while six focused on TMS, the results showed that tDCS significantly improved empathy quotient (EQ) and facial emotion recognition and processing (FERP) scores for emotions that conveyed a threat, and social and health/behavioral domains of autism treatment evaluation checklist (ATEC) also improved significantly. Active deep rTMS significantly reduced social relating impairments as measured by the Ritvo Autism Asperger Diagnostic Scale (RAADS) and decreased self-oriented anxiety in difficult social environments as measured by the interpersonal reactivity index (IRI), as compared to sham stimulation. However, the problems at this stage are that the sample size of clinical research is small and the types of trials are different, and the quality of literature is uneven, resulting in the inconsistent interpretation of research results. Therefore, it’s necessary to comprehensively and systematically evaluate the curative effect of the NIBS method on ASD patients. We conduct a rigorous systematic review and meta-analysis of related clinical randomized controlled trials (RCT). Looking for Evidence-Based Medicine Evidence of Objective Science, to provide decision-making and basis for the clinical rehabilitation treatment of NIBS method.

## 2. Methods

This systematic review and meta-analysis strictly followed the protocol developed by Preferred Reporting Items for Systematic Reviews and Meta-Analysis (PRISMA) guidelines (32) and is registered with PROSPERO (reference number: CRD42022366000).

### 2.1. Inclusion and exclusion criteria

Inclusion Criteria: ① Subjects: Clinically diagnosed autistic patients, regardless of race or gender; ② Intervention method: The intervention group was treated with TMS or tDCS; ③ Control group: sham stimulation, conventional treatment or blank control; ④ Study type: Randomized controlled trial. Include placebo (sham) control, baseline control, or candidate control; ⑤ Published in English.

Exclusion Criteria: ① Self before and after-control studies, cohort and case–control studies, cross-sectional studies, and other non-RCT; ② Literatures with no comparable baseline or no baseline reported; ③ Literature with imprecise design or inappropriate statistical methods; ④ Literatures with incomplete data, whose original data and the full text cannot be obtained after contacting the author; ⑤ Literatures with no corresponding outcome indicators; ⑥ Literatures with unclear diagnostic criteria, intervention time, and intervention programs; ⑦ Duplicate publications; ⑧ Conference abstracts, animal experiments, experimental protocols, expert experience summaries, case reports, meta-analysis, and review literature, etc.

### 2.2. Search strategy

Combination of computer and manual retrieval, from the establishment of the database to October 2022, the database includes PubMed, Web of Science, Medline, Embase, and Cochrane Library. Collect all RCTs of NIBS improving ASD, and supplement the literature by reading relevant reviews and references, etc. According to the way the combination of medical subject terms and free words, the retrieval time is from the establishment of the database to October 2022. Taking PubMed as an example, the retrieval strategy:#1: “autism spectrum disorder” [MeSH] OR autism spectrum disorders OR autism OR autistic spectrum disorder OR autistic spectrum disorders；#2: “transcranial direct current stimulation” [Mesh] OR “transcranial magnetic stimulation” [Mesh] OR repetitive transcranial magnetic stimulation OR noninvasive brain stimulation OR non-invasive brain stimulation OR transcranial electrical stimulation OR rTMS OR tDCS OR TMS OR NIBS；#3: “randomized controlled trial” [MeSH] OR random OR random allocation OR RCT；#4:#1 and #2 and #3.

### 2.3. Study selection and data extraction

The literature was collected, read, screened, and extracted according to the principle of independent extraction by two persons, Extract contents include ① Basic characteristics of the included literature: author, year, sample size, intervention measures, time, stimulus parameters, and outcome indicators, etc.; ② Key points related to biased risk assessment of literature; ③ The specific data of the outcome indicators.

### 2.4. Outcomes

Rehabilitation outcome indicators for patients with autism are mainly assessed by using graded scales or clinically set scales, Using continuous variables (mean and standard deviation) as the basis for symptom classification, including the following 4 parts: ① Autism Behavioral Checklist (ABC): The ABC is a behavior questionnaire which is completed by child’s parents or caregivers. The questionnaire marks five aspects of the child on a 4-point scale (including sensory, relating, body concept and object use, language, social, and self-care), ranging from 0 (no problem) to 3 (severe problem). The higher the score, the more serious the problem. The cutoff score was 49, and a score above 49 points indicated a high probability of ASD (33, 34). ② Autism Treatment Evaluation Checklist (ATEC): The ATEC is a self-administered questionnaire completed by the patient’s parents, teachers, or caregivers and consists of 77 items divided into 4 subtests. The first assesses speech or language communication with comprises 14 items. The second assesses sociability with 20 items. The third assesses sensory or cognitive awareness with 18 items. The fourth assesses the health/physical/behavior with 25 items. Score range, 0–179. A higher score indicates more serious symptoms of ASD (35). ③ Childhood Autism Rating Scale (CARS): The CARS is a tool that incorporates information from the caregiver’s report and direct observation from Clinicians, completed by clinicians, A score of ≥30 points indicates a possible diagnosis of ASD (36). ④ Repetitive Behavior Scale-Revised (RBS-R): The RBS-R was intended for use in evaluating repetitive behaviors observed in ASD primarily. It is a comprehensive 44-item parent/caregiver report questionnaire. The scale measures stereotyped behavior, self-injurious behavior, compulsive behavior, routine behavior, sameness behavior, and restricted behavior. Each item score range 0–4.The higher the score, the more frequently the behavior occurs (37).

### 2.5. Assessment of the risk of bias in the included studies

Two researchers assessed the bias risk of the included RCT according to the RCT bias risk assessment tool recommended by the Cochrane system and cross-checked the results (38). The content of the evaluation includes the following six aspects: ① Random allocation method; ② schemes of Allocation concealment; ③ Blinding of participants, personnel, and outcome assessment; ④ Completeness of outcome data; ⑤ Selective reporting; ⑥ Other bias. The risk of inclusion in the literature is divided into three levels: low bias risk, bias ambiguity risk, and high bias risk. If there are differences, resolve them through discussion. If no agreement can be reached, consult the third author. In addition, funnel charts of major outcome indicators were evaluated to assess publication bias.

### 2.6. Statistical analyses

RevMan 5.3 software (computer program, version 5.3, Copenhagen: The Nordic Cochrane Centre, The Cochrane Collaboration, 2014) was used for statistical analysis. Since the included studies are different in terms of intervention measures, measurement methods, and outcome assessment, SMD and its 95% CI were selected for the heterogeneity test for the combined effect value. If *p* > 0.1, I^2^ < 50%, it can be considered that the included studies are homogeneous, and the fixed effect model should be used for meta-analysis; If *p* > 0.1, I^2^ < 50%, it can be considered that the included studies are homogeneous, and the fixed effect model should be used for meta-analysis; If the data with research results cannot be meta-analyzed, only descriptive analysis will be performed. Because the results of the random effect model are more conservative, to ensure that the data results are more credible, this study adopts the random effect model, and all analyses calculate 95% confidence intervals.

## 3. Results

### 3.1. Literature search results

**Figure 1:**
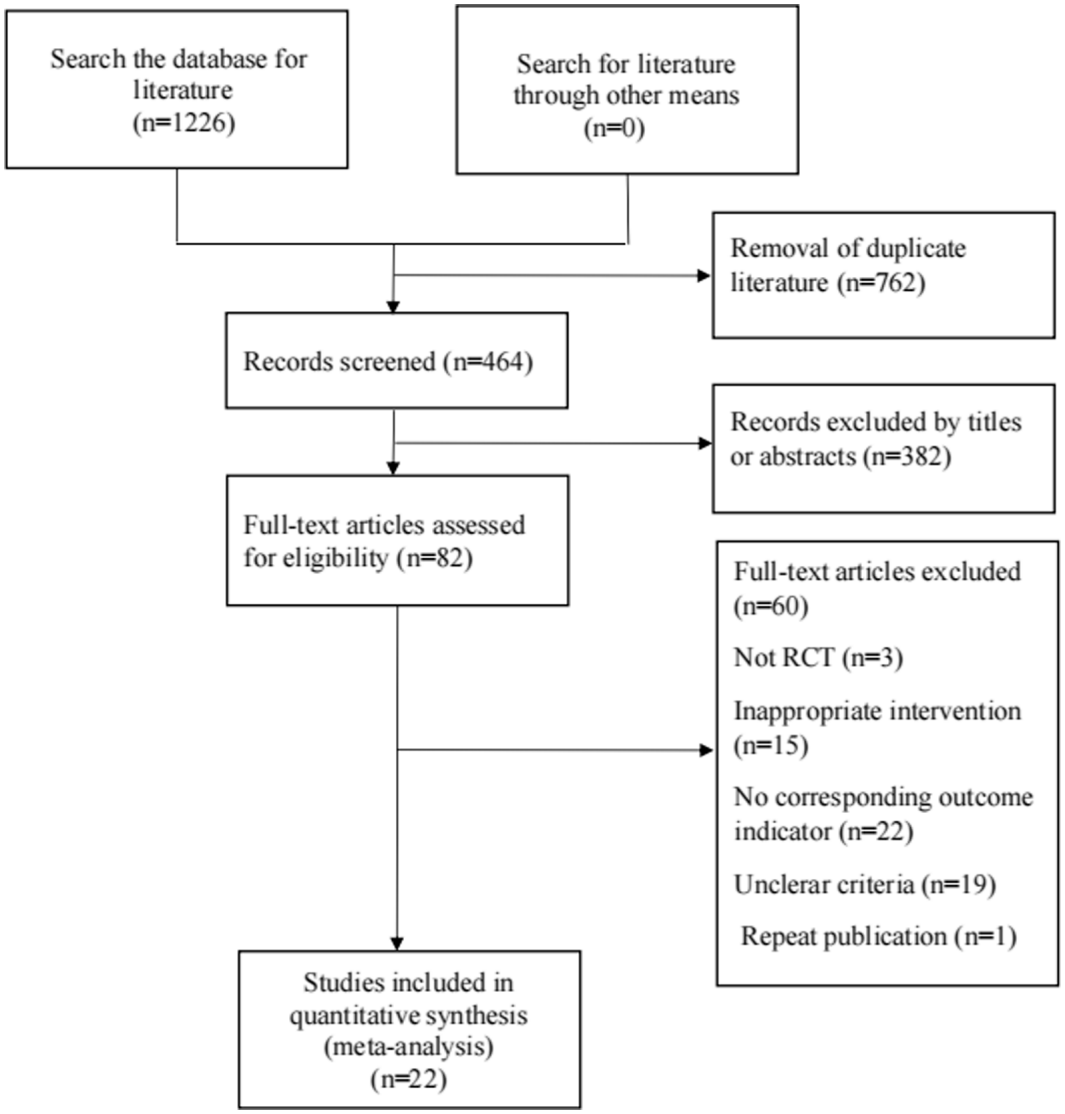
Literature selection process.

Figure 1 shows the flow diagram for the selection of the included studies. 1,226 related literature were obtained in the preliminary examination. After removing duplicate publications by EndnoteX9, there are 464 articles. After reading titles and abstracts, 382 articles were removed. After reading the full text of the remaining 82 articles, 22 articles were finally included. A total of 25 RCTs were included in 22 articles.

### 3.2. Study characteristics

Tables 1–3 show the characteristics, technical parameters, and results of the included studies. In this study, one of the 22 studies included three RCTS (39), so in the end we selected 25 RCTs studies using TMS or tDCS to treat ASD patients through standard and rigorous screening procedures. Ten studies used tDCS (40–49) and 15 studies used TMS (19, 39, 50–59) as a treatment tool. Among the included articles, 19 controlled studies used a sham stimulation group as a control group, and the remaining 5 (50–54) compared patients with autism with patients in a waiting group. Among included studies, a total of 829 ASD patients were treated with NIBS (305 patients received tDCS intervention and 521 patients received TMS intervention). Four studies (19, 55–57) recruited adults with ASD and all focused on TMS intervention studies, the remaining studies mainly recruited children and adolescents with ASD. Subjects spanned virtually the entire autism spectrum, including high-functioning (101 patients), low-functioning (35 patients), and Asperger’s with and without language and cognitive impairments (35 patients). There are large differences between the various treatment options. In tDCS treatment studies, the common stimulation area is the DLPFC. In most cases, the left hemisphere is preferred for anodal or cathodal stimulation. The anodal is the most commonly selected stimulation method in the study, and the intensity is often selected for 1 to 1.5 mA. The treatment lasted 20 min and the frequency of treatment varied from once a day to twice a week. Of these, 8 studies (40–42, 45–49) looked at behavioral improvements in patients with autism as the primary outcome indicator, The Autism Treatment Evaluation Checklist (ATEC) and the Aberrant Behavior Checklist (ABC) are the most commonly used tools for post-treatment evaluation. In addition, 4 studies (40–43) used more objective neurobiological markers as the outcome of the measurement, focusing on patients’ neuropsychological function, brain connectivity, and spontaneous, rhythmic electrical activity of brain cell groups. At present, we can see that patients showed significant improvement after active tDCS stimulation. Of all the included studies, only one study (44) reported mild side effects, which disappeared soon after discontinuation of treatment.

**Table 1:** Study and sample characteristics for included studies.

Author	Country	Study design	Experimental group				Control group				
			Number	Male	Female	Age (years)	Number	Male	Female	Age (years)	Diagnosis
Qiu (2021)	China	RCT (sham controlled)	20	16	4	-	20	14	6	-	ASD
Hadoush (2020)	Jordan	RCT (sham controlled)	25	19	6	7.6 ± 2.2	25	22	3	8.0 ± 2.8	ASD
Salehinejad (2021)	Iran	RCT (sham controlled)	7	-	-	10.7 ± 1.9	7	-	-	10.7 ± 1.9	ASD
Zemestani (2022)	Iran	RCT (sham controlled)	17	-	-	-	15	-	-	-	ASD
Amatachaya (2014)	Thailand	RCT (sham controlled)	10	10	0	6.4 ± 1.1	10	10	0	6.4 ± 1.1	ASD
Amatachaya (2015)	Thailand	RCT (sham controlled)	10	10	0	6.4 ± 1.1	10	10	0	6.4 ± 1.1	ASD
Sun (2022)	China	RCT (sham controlled)	19	15	4	8.0 ± 1.9	18	15	3	8.0 ± 1.9	ASD
Mahmoodifar (2020)	Iran	RCT (sham controlled)	9	-	-	11.04 ± 2.80	9	-	-	9.31 ± 2.70	ASD
Han (2021)	China	RCT (sham controlled)	20	18	2	17.03 ± 2.55	21	20	1	17.10 ± 2.30	ASD
Hadoush (2022)	Iran	RCT (sham controlled)	18	15	3	8.1 ± 2.9	18	16	2	7.6 ± 2.6	ASD
Joshua (2010)	America	RCT (waitlist controlled)	16	-	-	13.9 ± 5.3	9	-	-	13.5 ± 2.0	ASD
Panerai (2014) study I	Italy	RCT (sham controlled)	9	-	-	13.56 ± 1.83	-	-	-	13.56 ± 1.83	Low-function ASD
Panerai (2014) study II	Italy	RCT (sham controlled)	12	-	-	13.56 ± 1.88	5	-	-	13.24 ± 2.95	Low-function ASD
Panerai (2014) study III	Italy	RCT (sham controlled)	6	-	-	16.13 ± 3.11	-	-	-	16.13 ± 3.11	Low-function ASD
Panerai (2014) study IV	Italy	RCT (training controlled)	8	-	-	13.27 ± 4.03	5	-	-	14.17 ± 4.24	Low-function ASD
Ni (2021)	China	RCT (sham controlled)	40	35	5	13.0 ± 2.8	35	30	5	12.5 ± 2.9	ASD
Kang (2021)	China	RCT (sham controlled)	16	13	3	7.8 ± 2.1	16	13	3	7.2 ± 1.6	ASD
Ni (2017)	China	RCT (sham controlled)	19	-	-	20.8 ± 1.4	19	-	-	20.8 ± 1.4	ASD
Iska (2021)	Canada	RCT (sham controlled)	16	13	3	23.1 ± 4.66	12	8	4	23.4 ± 4.93	ASD
Peter (2014)	Israel	RCT (sham controlled)	15	13	2	33.87 ± 13.07	13	10	3	30.54 ± 9.83	High-function ASD, Asperger
Stephanie (2020)	Canada	RCT (sham controlled)	20	14	6	23.50 ± 4.2	20	14	6	21.65 ± 4.6	ASD
Sokhadze (2009)	America	RCT (waitlist controlled)	8	8	0	18.3 ± 4.8	5	5	0	16.2 ± 5.7	ASD
Casanova (2012)	America	RCT (waitlist controlled)	25	-	-	12.9 ± 3.1	20	-	-	13.1 ± 2.2	ASD
Sokhadze (2012)	America	RCT (waitlist controlled)	20	16	4	13.5 ± 2.5	20	16	4	14.1 ± 2.4	ASD, Asperger
Sokhadze (2018)	America	RCT (waitlist controlled)	86	71	15	13.1 ± 1.78	26	22	4	13.3 ± 1.78	High-function ASD

**Table 2:** Stimulation parameters for included studies.

Study	Interventions		tDCS procedure				TMS procedure				Treatment duration
	E	C	Polarity	Anodal location	Cathodal location	Intensity (mA)	Target location	Frequency (Hz)	MT (%)	Pulses	
Qiu (2021)	tDCS	Sham	Anodal	Left DLPFC	Right shoulder	1					15 × 20 min (five times a week)
Hadoush (2020)	tDCS	Sham	Bilateral anodal	Left and right frontocentral (FC1-FC2)	Left and right supraorbital (Fp1-Fp2)	1					10 × 20 min (five times a week)
Salehinejad (2021)	tDCS	Sham	Anodal	1.Right tem- poroparietal junction (CP6) 2.vmPFC (Fpz)	Left shoulder	1					20 min (3 single sessions)
Zemestani (2022)	tDCS	Sham	Anodal	Left DLPFC	right DLPFC	1.5					10 × 15 min (two times a week)
Amatachaya (2014)	tDCS	Sham	Anodal	Left DLPFC	Right shoulder	1					10 × 20 min (last 8 weeks)
Amatachaya (2015)	tDCS	Sham	Anodal	Left DLPFC	Right shoulder	1					20 min (last 3 weeks)
Sun (2022)	tDCS + rehabilitation	Sham + rehabilitation	Anodal	Left DLPFC	Right supraorbital	1.5					12 × 20 min (three times a week)
Mahmoodifar (2020)	tDCS	Sham	Anodal	Left motor cortex (M1)	Right supraorbital	1.5					10 × 20 min
Han (2021)	tDCS + cognitive training	Sham + cognitive training	Anodal	Left DLPFC	Right supraorbital	1					10 × 20 min (last 2 weeks)
Hadoush (2022)	tDCS	Sham	Bilateral anodal	Left and right cerebellar hemispheres	left and right supra-orbital area	1					10 × 20 min (five times a week)
Joshua (2010)	rTMS	Waitlist					Left and right DLPFC	1	90	150 (15 × 10)	per week (last 12 weeks)
Panerai (2014) study I	rTMS	Sham					Left and right premotor cortex,2.5 cm rostral to primary motor cortex	LFrTMS: 1; HFrTMS: 8	90	LFrTMS: 900; HFrTMS: 30 trains of 30 stimuli each trial lasting 3.6 s	Every 2 weeks
Panerai (2014) study II	rTMS	Sham					Left premotor cortex, 2.5 cm rostral to primary motor cortex	LFrTMS: 1; HFrTMS: 8	90	LFrTMS: 900; HFrTMS: 30 trains of 30 stimuli each trial lasting 3.6 s	Every weekday (10 days), over 2 weeks
Panerai (2014) study III	rTMS	Sham					Left premotor cortex, 2.5 cm rostral to primary motor cortex	LFrTMS: 1; HFrTMS: 8	90	LFrTMS: 900; HFrTMS: 30 trains of 30 stimuli each trial lasting 3.6 s	Daily (last 5 days)
Panerai (2014) study IV	rTMS	Eye–hand integration training					Left premotor cortex, 2.5 cm rostral to primary motor cortex	8	90	LFrTMS: 900; HFrTMS: 30 trains of 30 stimuli each trial lasting 3.6 s	Every weekday (10 days), over 2 weeks
Ni (2021)	iTBS	Sham					Bilateral pSTS	50	80	38,400	twice a week (last 4 weeks)
Kang (2021)	rTMS	Sham					Left, right and bilateral DLPFC	1	90	180 (18 × 10)	twice a week (last 9 weeks)
Ni (2017)	iTBS	Sham					Left and right DLPFC; posterior superior temporal sulcus	50	80	Two courses of 600 on each hemisphere, left first, 5 min apart	1 week interval between sessions
Iska (2021)	rTMS	Sham					Bilateral DLPFC	20	90	1,500	five times a week (last four weeks)
Peter (2014)	rTMS	Sham					Bilateral DMPFC, coil centered and 7 cm anterior to M1, 3–4 cm from nasion	5	100	1,500	Every weekday (10 days)
Stephanie (2020)	rTMS	Sham					Left and right DLPFC	20	90	1,500	20-session (last 4 weeks)
Sokhadze (2009)	rTMS	Waitlist					Left DLPFC, 5 cm anterior to maximal FDI response	0.5	90	150 (15 × 10)	twice a week (last 3 weeks)
Casanova (2012)	rTMS	Waitlist					Left and right DLPFC	1	90	150 (15 × 10)	once a week (last 12 weeks)
Sokhadze (2012)	rTMS	Waitlist					Left and right DLPFC	1	90	150 (15 × 10)	once a week (last 12 weeks)
Sokhadze (2018)	rTMS	Waitlist					Left and right DLPFC	1	90	180 (9 × 20)	once a week (last 18 weeks)

E, experimental group; C, control group; MT, Motor threshold; tDCS, transcranial direct current stimulation; HFrTMS, High-frequency repetitive transcranial magnetic stimulation; iTBS, Intermittent Theta-burst stimulation; LFrTMS, Low-frequency repetitive transcranial magnetic stimulation; rTMS, Repetitive transcranial magnetic stimulation; RCT, randomized controlled trial; DLPFC, dorsolateral prefrontal cortex; DMPFC, dorsomedial prefrontal cortex; FDI, First Dorsal Interosseous; vmPFC/Fpz, ventromedial prefrontal cortex; Fp1, left supraorbital area; Fp2, right supraorbital area; M1, left primary motor cortex; CP6/rTPJ, right temporoparietal junction; FC1/FC2, left and right frontocentral regions; pSTS, posterior superior temporal sulcus.

**Table 3:** Outcome measures and results for included studies.

Study	Cognitive measures	Behavioral measures	Biological measures	Cognitive outcomes	Behavioral outcomes	Biological outcomes	Follow up	Side effects
Qiu (2021)		Key symptoms (CARS, ABC, RBS-R), sleep condition (CSHQ)			Real tDCS significantly reduced CARS and sleep habit scores, while sham tDCS significantly reduced ABC scores			None
Hadoush (2020)		Symptoms (ATEC)			Significant potential therapeutic effects on children with ASD in terms of improvements in sociability, behavior, health, and physical conditions			None
Salehinejad (2021)	Theory of mind test (TOM)			Compared with rTPJ tDCS and sham stimulation, anodal vmPFC tDCS significantly improved ToM in children with ASD				Mild adverse effects
Zemestani (2022)	Theory of Mind (ToM)	Gilliam Autism Rating Scale-second edition (GARS-2),Emotion Regulation Checklist (ERC)			A significant improvement of autism symptom severity, theory of mind, and emotion regulation strategies was observed for the active as compared to the sham stimulation group			None
Amatachaya (2014)	CGI-I	CARS, ATEC, CGAS			Anodal F3 tDCS Improved social functioning, behavior, sensory or cognition, ATEC scores compared to sham tDCS			None
Amatachaya (2015)		ATEC	EEG record		Improvement in social behavior and behavioral ATEC scores after receiving the tDCS intervention	PAF significantly increased at the stimulation site		None
Sun (2022)		ABC	Electroencephalography		Behavioral abilities improved significantly in both groups after receiving the intervention. The active tDCS group was significantly better than the control group.	MMN amplitude was elevated between both groups, but there was no significant difference		None
Mahmoodifar (2020)		Movement Assessment Battery for Children-2 (MNBC-2)			sham tDCS combined with motor training improved balance. Active tDCS + training showed a significantly higher improvement compared to sham + training			None
Han (2021)		Social functioning (SRS-2)	Measured prefrontal resting-state functional connectivity (rsFC)		improvement in overall social functioning in the active and sham tDCS groups differed significantly	Greater interindividual variability among participants in rsFC raw change in the right medial PFC		None
Hadoush (2022)			Record and calculate the approximate entropy (ApxEnt) values of the resting-state electroencephalograph (EEG) data obtained from a 64-channel EEG system			Bilateral cerebellar anodal tDCS modulated and increased the brain complexity in children with ASD		None
Joshua (2010)	Reaction time and error rates in an oddball- type task	ABC, RBS-R, SRS	Gamma activity	No significant differences	Significant decrease in irritability and repetitive behavior subscales of ABC, and in repetitive behavior subscale of RBS-R after treatment	Increased gamma power to targets and decreased gamma power to non-targets after treatment		Itching sensation at nose, mild headache
Panerai (2014) study I	Degree of completion of hand-eye combination tasks from PER-P			HFrTMS: increase in eye–hand integration after TMS to left premotor cortex. LFrTMS and sham: no differences in eye–hand integration.				None
Panerai (2014) study II	Degree of completion of hand-eye combination tasks from PER-P			LFrTMS, HFrTMS, and sham: highest increase in mean performance with HFrTMS, followed by LFrTMS and sham. Pre-post comparisons showed difference only for HFrTMS				None
Panerai (2014) study III	Degree of completion of hand-eye combination tasks from PER-P			Significant increase in eye-hand integration after TMS, in comparison to sham			2,5 days follow up; HfrTMS showed no difference from sham TMS or from baseline assessment	None
Panerai (2014) study IV	Degree of completion of hand-eye combination tasks from PER-P			Pairwise comparisons showed a statistical difference between HFrTMS + Eye–hand integration training and both treatments alone			1 month follow up; TMS + training significantly better than either intervention alone after 1 week; at 2 weeks TMS + training superior to training alone; at 4 weeks no differences between groups	None
Ni (2021)	Reading the Mind in the Eyes test (RMET), Frith–Happe animations task	SRS, RBS-R		no significant group-by-time interaction	no significant group-by-time interaction			Slight headache, dizziness, tinnitus, and anxiety
Kang (2021)		ABC	Recurrence quantification analysis (RQA) was employed to quantify the nonlinear features of electroencephalogram (EEG) signals recorded during the resting state. Three RQA measures, including recursive rate (RR), deterministic (DET) and mean diagonal length (L) were extracted from the EEG signals to characterize the deterministic features of cortical activity.		Significant improvements in ABC scores in social relating behaviors for the experimental group	Significant differences in RR and DET were observed between the experimental group and the control group.		None
Ni (2017)	CCPT, WCST	Y-BOCS, SRS		Significant decrease in reaction time in the CCPT after DLPFC stimulation compared to sham	Comparison to sham, significant reduction in compulsive behaviors subscale of Y-BOCS after pSTS stimulation, and improvement in social communication subscale of SRS after DLPFC stimulation			Transient muscle twitches around the eyes
Iska (2021)			Examined glutamatergic (Glx) or γ-aminobutyric acid (GABA) metabolite levels			Active rTMS can modulate Glx levels in individuals with ASD, and that the direction of change is associated with baseline Glx levels.		None
Peter (2014)	Reading the mind in the eyes test and mentalizing test	RAADS, AQ, IRI		No significant differences in mentalizing measures	Significant decrease in social relatedness subscale of RAADS, and in personal distress subscale of IRI compared to sham			light headedness and facial discomfort during stimulation
Stephanie (2020)	Cambridge Neuropsychological Test Automated Battery (CANTAB) and BRIEF Metacognition Index (BRIEF-MCI)			No significant difference between active vs. sham rTMS on executive functions performance				Mild and transient discomfort
Sokhadze (2009)	Reaction time and error rates in an oddball- type task	ABC; RBS-R; SRS; CGI	Gamma activity; ERPs	No significant differences in reaction time and error rates after treatment	Significant decrease in repetitive behavior of RBS-R	Decrease in gamma power, amplitude of the frontal P3a and latency of the centro-parietal P3b to non-targets after treatment		None
Casanova (2012)	Reaction time and error rates in an oddball- type task	ABC; RBS-R; SRS	ERPs	Significant decrease in total error and omission error rates after treatment	Significant decrease in irritability subscale of ABC, and in repetitive behavior subscale of RBS-R after treatment	Increased amplitude of the frontal and parietal N200 and frontal P3a and reduced latency of the frontal N200 to targets after treatment		None
Sokhadze (2012)	Reaction time and error rates in an oddball- type task		ERPs	Slowing of post-error reaction time in TMS group compared to waiting list, and significant decrease in omission error rate after treatment		Increased amplitude and reduced latency of ERN component after treatment		None
Sokhadze (2018)	Reaction time and error rates in an oddball- type task	ABC, RBS-R	ERPs	Lower percentage of committed errors, slower latency of commission errors	Decreased of T-score of the RBS-R after 18 sessions of rTMS, along with decreased irritability, lethargy/social withdrawal and hyperactivity rating scores of the ABC questionnaire.	Restored normative post-error reaction time slowing in both early and later-stage ERP indices, enhanced magnitude of error-related negativity (ERN), improved error monitoring and post-error correction functions		None

CARS, Childhood Autism Rating Scale; ABC, Aberrant Behavior Checklist; RBS-R, Repetitive Behavior Scale-Revised; CSHQ, Children’s Sleep Habits Questionnaire; ATEC, Autism Treatment Evaluation Checklist; TOM, theory of mind test; GARS-2, Gilliam Autism Rating Scale-second edition; ERC, Emotion Regulation Checklist; CGAS, Children’s Global Assessment Scale; CGI-I, Clinical Global Impression-Improvement; F3, left dorsolateral prefrontal cortex; EEG, Electroencephalography; PAF, peak alpha frequency; MMN, mismatch negativity; MNBC-2, Movement Assessment Battery for Children-2; SRS-2, Social Responsiveness Scale-2nd; CCPT, Conner’s Continuous Performance Test; WCST, Wisconsin Card Sorting Test; Y-BOCS, Yale-Brown Obsessive Compulsive Scale; RAADS, Ritvo Autism-Aspergers Diagnostic Scale; AQ, Autism Spectrum Quotient; IRI, Interpersonal Reactivity Index; ERP, event-related potential.

In the rTMS study, 2 studies (55, 58) used iTBS for clinical intervention. The remaining 13 studies used rTMS traditionally. Most studies have applied unilateral or bilateral stimulation to DLPFC using low-frequency stimulation (0.5–1 Hz). An article (44) delivered multisegmental stimulation to the bilateral premotor cortex (PrMC) at a stimulation frequency of 1–8 Hz. 1 study (47) implemented rTMS targeting the dorsal medial prefrontal cortex at a stimulation frequency of 5 Hz. It is worth noting that Ni et al. (58) chose the posterior superior temporal sulcus (posts) (60), one of the three target stimulation areas currently recognized by the academic community as promising to improve the core symptoms of ASD, as the target for treatment with 50 Hz iTBS. The results showed that cognitive improvement was not significant in a patient with autism, which may be related to individual signs and the length of treatment.

Treatment schedules also varied widely, with most of the included studies receiving daily or twice-weekly treatments for a minimum of 5 days and a maximum of 12 weeks. Only two studies used neuronavigation to guide stimulation of the intended cortical region, other common alternatives are EEG positioning or recommended by the developers of the coil. The most common side effects were mild headache and skin irritation, but the symptoms were mild.

### 3.3. Risk of bias of included studies

**Figure 2:**
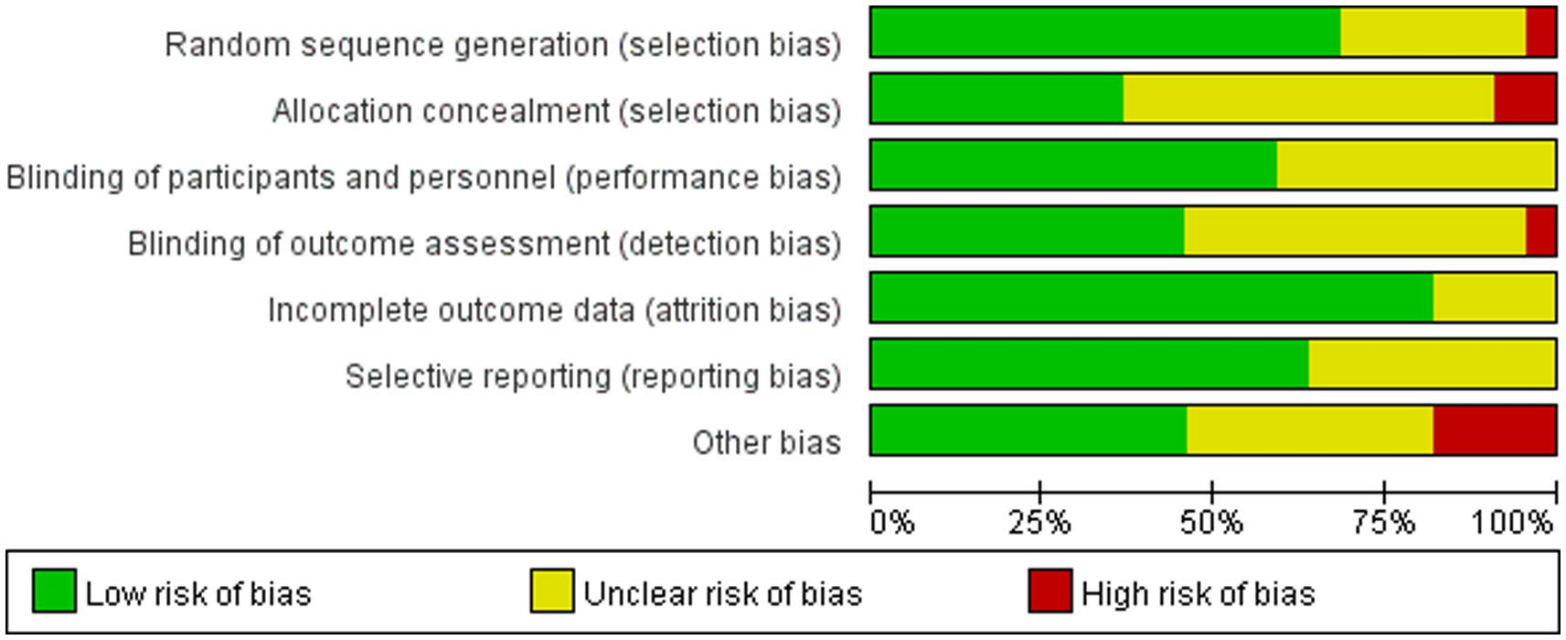
Risk of bias of the included studies.

**Figure 3:**
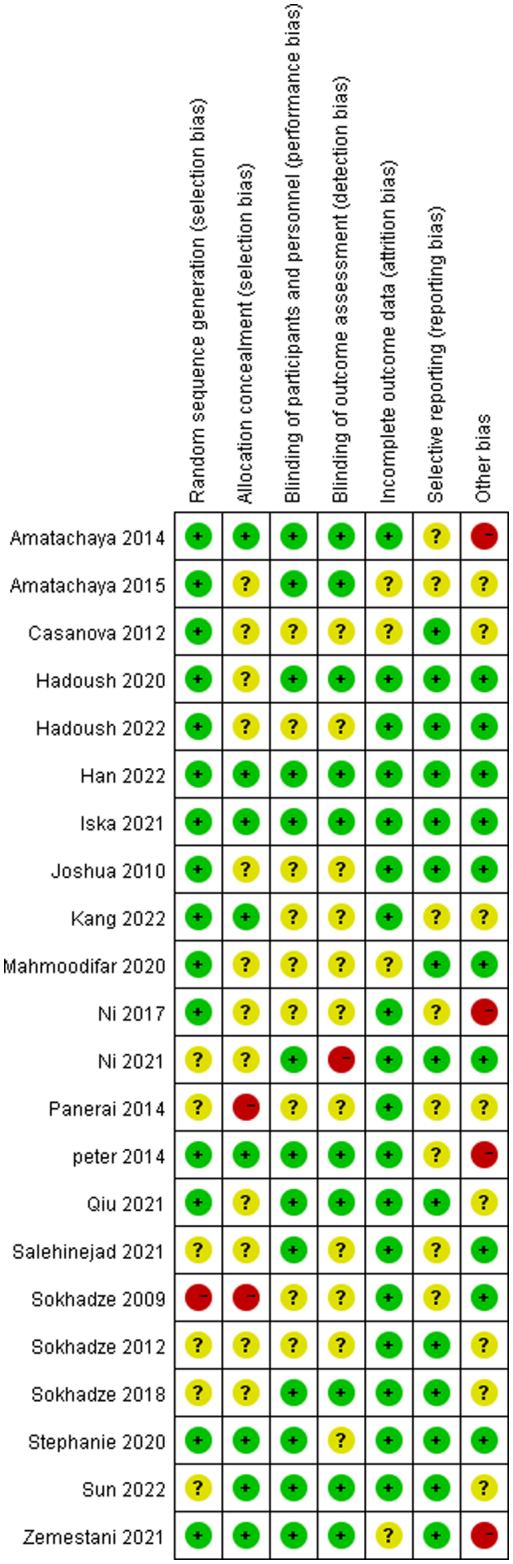
Risk of bias summary of the included studies.

**Figure 4:**
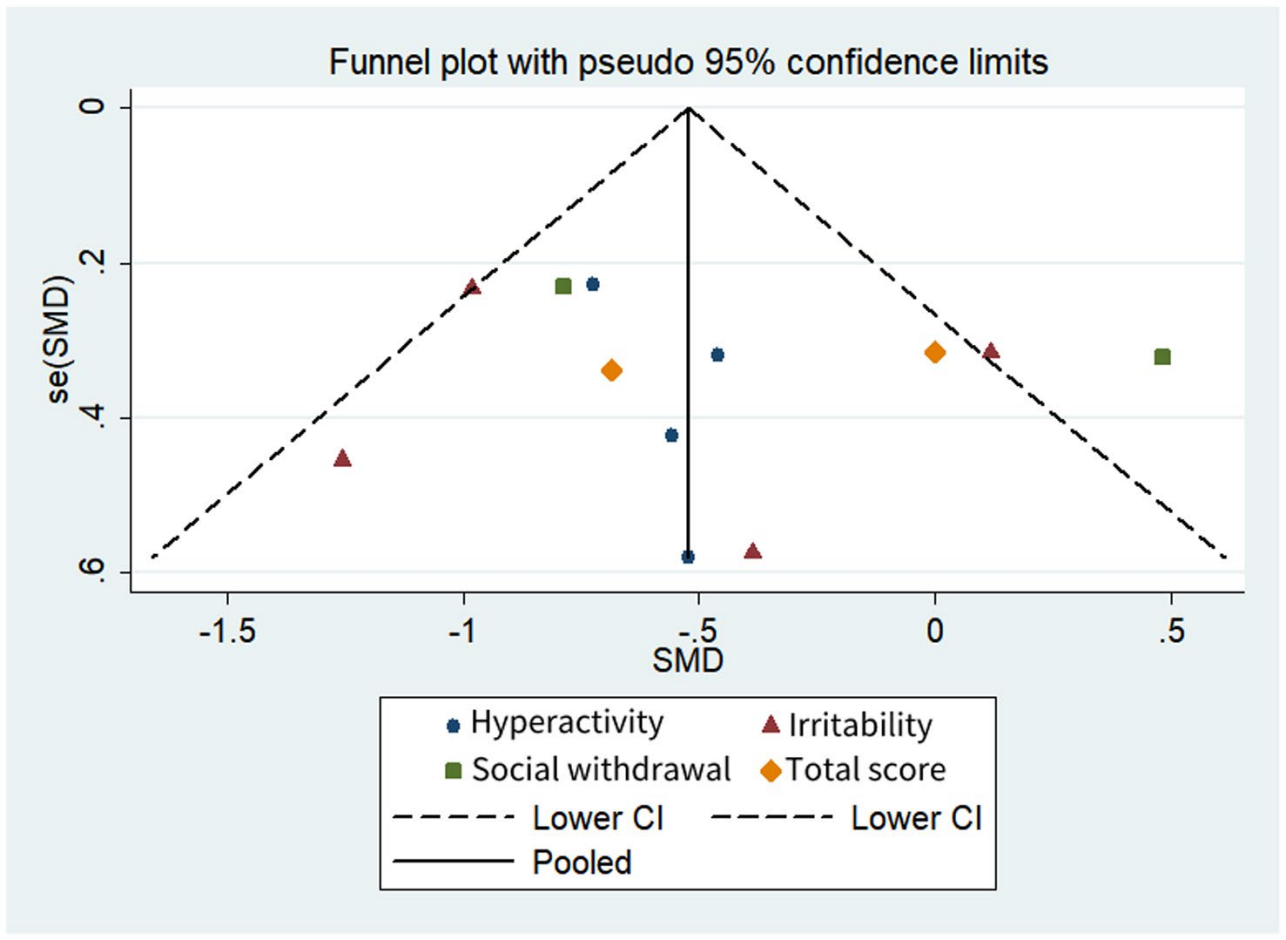
Funnel plot comparison of the ABC scores of the active and control groups. Asymmetries were not observed.

Table 3 and Figures 2, 3 shows a summary of the quality assessment of the selected studies. Details of bias risk in all included studies are shown in Figures 2, 3. The included studies were evaluated using the Cochrane Bias Risk Assessment Scale. All 18 studies reported subjects at baseline and were comparable; The literature refers to “randomization” or “randomized controlled trials,” 14 described specific random methods, such as random number table and computer random, and 6 carried out Allocation concealment. Thirteen mentioned signing informed consent. In allocation concealment, only 2 studies were high risk and 8 studies were low risk. For the blind approach to outcome assessment, only 1 study had a high risk and 11 had an undefined risk of bias. Due to the nature of the NIBS intervention, all studies are not free from implementation bias. Analysis of funnel plot using the ABC scale score as the outcome indicator (Figure 4). It shows that the distribution of scattered points on both sides of the midline is basically symmetrical, showing an inverted funnel shape, the possibility of bias in the included literature is small, and the meta-analysis results are more reliable. Because the number of included literature using ATEC, CARS, and RBS-R as indicators is small, it is not suitable for funnel plots.

### 3.4. Meta-analysis results

Of the 22 included articles, 11 had extractable data for a meta-analysis of behavioral or cognitive outcomes in patients treated with NIBS. In the process of data extraction, we found that the evaluation indicators used in some literature are different, which makes it impossible to combine the data for analysis. So we chose ABC, ATEC, CARS, and RBS-R which are commonly used internationally, as indicators for evaluating the behavior and cognitive abilities of autistic patients for analysis.

#### 3.4.1. ABC

A total of 5 studies (41, 45, 50, 51, 54) were included, and 4 literature (17, 34, 35, 38) reported the score of the hyperactivity subscale, Meta-analysis results of the random effects model showed that the score of the experimental group was lower than that of the control group, there was a significant difference (SMD = −0.6, 95%CI [−0.93, −0.28], *p* < 0.01). The heterogeneity test showed no significant heterogeneity among different studies (χ^2^ = 0.55, *p* = 0.91, I^2^ = 0% < 50).

Four articles (45, 50, 51, 54) reported irritability subscale scores and meta-analysis of the random-effects model showed no significant difference between the test group and the control group (SMD = −0.61, 95%CI [−1.26, 0.04], *p* = 0.06). The heterogeneity mainly comes from the study of Qiu et al. (39), after removing outliers, I2 drops to 0. We found that Qiu et al. (45) studied autistic children aged 2–6 years, whereas the other three studies studied autistic adolescents aged 13–18. Therefore, the discrepancy may be due to different study subjects. Notably, although the difference between the test and control groups was not significant, there was a trend in favor of NIBS treatment.

**Figure 5:**
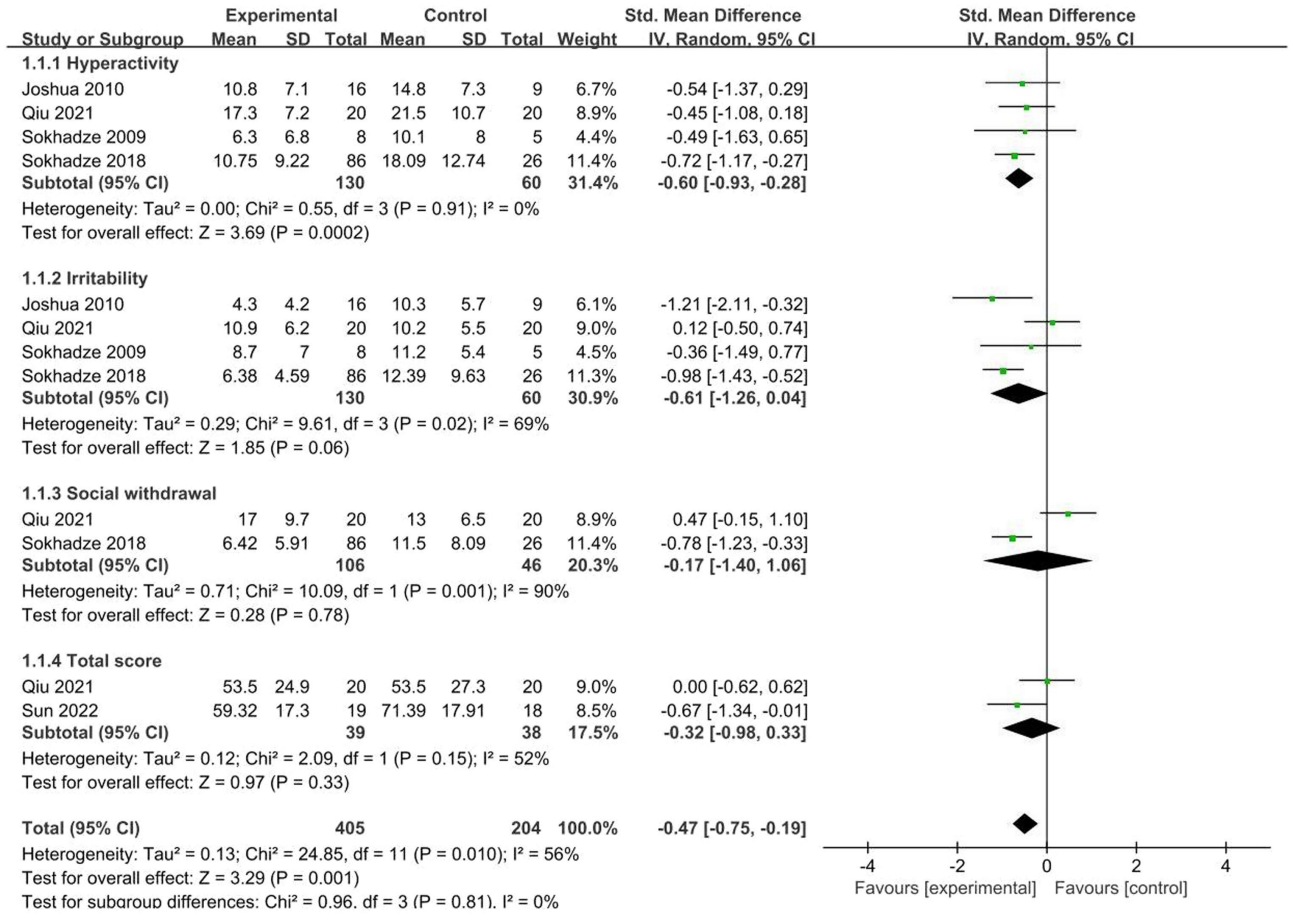
Forest plot of the effect of NIBS on ABC improvement.

Two literature (45, 54) reported the scores of the Social Withdrawal subscale, and the meta-analysis results of the random effect model showed that there was no significant difference between the scores of the test group and the control group (SMD = −0.71, 95%CI [−1.40, 1.06], *p* = 0.78). Two literature (41, 45) reported the total score of ABC, the meta-analysis results of the random effect model showed that there was no significant difference between the scores of the test group and the control group (SMD = −0.32, 95%CI [−0.98, 0.33], *p* = 0.33). But, due to the small number of studies included, the results should be interpreted with caution (Figure 5).

#### 3.4.2. ATEC

**Figure 6:**
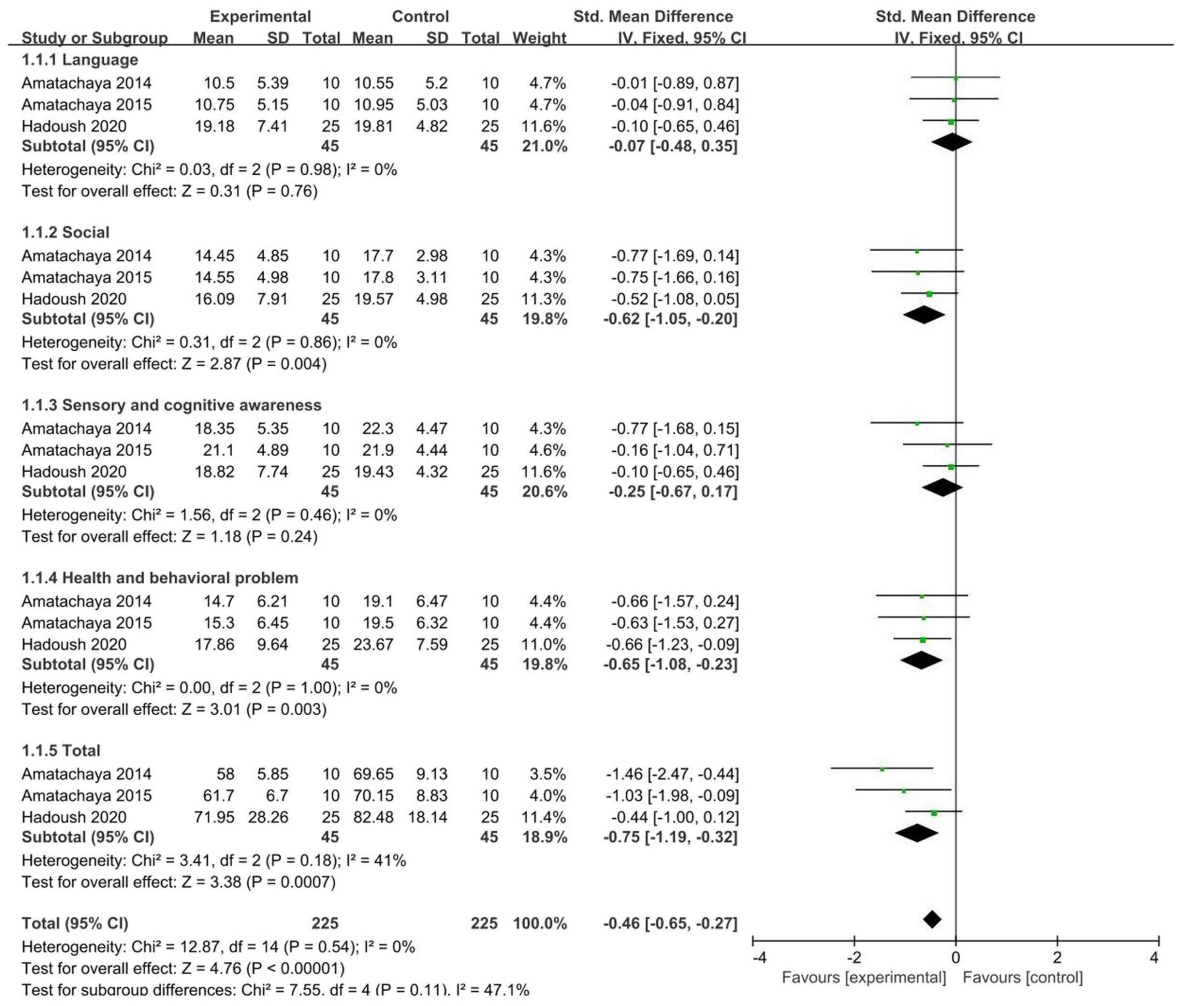
Forest plot of the effect of NIBS on ATEC improvement.

A total of 3 studies (46, 47, 54) were included, and meta-analysis was performed on the five dimensions of ATEC. The results showed that test groups are significant improvements in the three dimensions of ATEC: social (SMD = −0.62, 95% CI [−1.05, −0.20], *p* = 0.004), health and behavioral problems (SMD = −0.65, 95% CI [−1.08, −0.23], *p* = 0.003), and ATEC total score (SMD = −0.75, 95% CI [−1.19, −0.32], *p* < 0.001). The heterogeneity test showed no significant heterogeneity among studies (Figure 6).

#### 3.4.3. CARS

**Figure 7:**
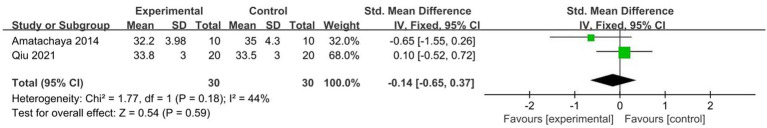
Forest plot of the effect of NIBS on CARS improvement.

A total of 2 studies (45, 46) were included, χ^2^ = 1.77, *p* = 0.18, I^2^ = 44% < 50%. The results showed no significant heterogeneity. Analysis using fixed effects model, the total combined effect shows: Combined effect value SMD = −0.14 (95%CI = −0.65, 0.37), the overall effect test *Z* = 0.54, *p* = 0.59, As seen in the forest plot, the combined effect size (black diamond) intersects the invalid vertical lines, showing no significant difference in CARS scores between the two groups. In other words, the NIBS did not have a significant effect on patients with ASD. But given the small number of studies included, the results should be treated with caution (Figure 7).

#### 3.4.4. RBS-R

**Figure 8:**
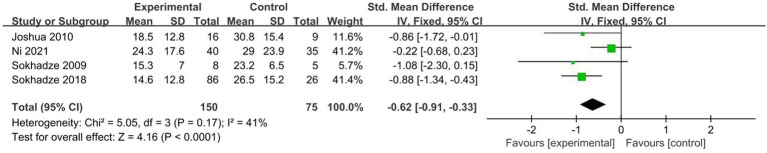
Forest plot of the effect of NIBS on RBS-R improvement.

A total of 4 studies (50, 51, 54, 58) were included, *χ*^2^ = 5.05, *p* = 0.17, I^2^ = 41% < 50%. The results showed no significant heterogeneity. Analysis using fixed effects model, the total combined effect shows: SMD = −0.62 (95%CI = −0.91, −0.33), the overall effect test *Z* = 4.16, *p* < 0.001. As seen in the forest plot. The combined effect size (black diamond) was located on the left side of the vertical line, indicating a significant difference in RBS-R scores between the two groups, indicating that NIBS significantly improved repetitive behavior in ASD patients (Figure 8).

## 4. Discussion

The purpose of this study was to comprehensively and systematically evaluate the clinical efficacy of NIBS in patients with ASD. To our knowledge, this is the first meta-analysis of NIBS treatment in ASD populations. These studies point to NIBS as a potential intervention to reduce autism-related symptoms and improving neuropsychological function in people with autism. This meta-analysis showed that NIBS improved behavioral and cognitive abilities in people with autism compared to controls. This result is consistent with the conclusion of the latest meta-analysis (61, 62).

Behavioral and cognitive abnormalities of ASD are often characterized by severe social difficulties and stereotyped behaviors. Neuropsychology suggests that this inappropriate behavior of ASD is related to executive dysfunction, which may lead to the weakened ability of patients with ASD to regulate their own behaviors, unable to restrain unconscious behaviors, and unable to learn new behaviors, thus being subject to their own stereotyped behaviors. Qiu et al. (45) explored the effect of tDCS on children with ASD under 7 years old, by placing the anode on the left DLPFC and the cathode on the right upper arm, giving tDCS for 1 mA, 20 min, 15 times in total. CARS, ABC, and RBS-R were used to evaluate before and after treatment. The results showed that the social interaction ability of children with ASD was improved and well tolerated, That tDCS is a promising therapeutic method. Amatachaya et al. (46) used anode of tDCS to intervene in the left DLPFC of ASD patients, and found that the stereotyped behavior improved. In addition, Han et al. (42) performed 10 times tDCS interventions on 41 adult patients with ASD, which also showed a good rehabilitation effect in terms of executive function. This suggests that tDCS can indeed improve executive dysfunction in patients of ASD, for reasons closely related to the DLPFC site. Because in the process of participating in executive function, DLPFC combines with striatum and other structures to form a loop structure, which is involved in executive function, problem-solving, cognitive function, etc. (62). In addition, the DLPFC is also the highest cortical area involved in motor planning, organization, and regulation/inhibition, and is closely related to other areas, such as the orbitofrontal cortex, thalamus, parts of the basal ganglia (especially the dorsal caudate nucleus) (63). This function and association also link the DLPFC to behavioral abnormalities such as restrictive and repetitive behaviors, hypersensitivity (overreaction), and blunted response to various stimuli (under response). Therefore, DLPFC is also regarded as the main target brain area for the treatment of ASD (45). Amir (64) and Nelson et al. (65) confirmed that tDCS intervention in DLPFC could improve working memory, attention, and vigilance, which indicated that tDCS could produce neuroregulatory effects on DLPFC, thus improving the cognitive function of patients with ASD. Other areas of the brain, the ventromedial prefrontal cortex (vmPFC)associated is also with ASD. Salehinejad et al. (44) stimulated the vmPFC region of ASD patients by giving anode of tDCS,1 mA, and evaluated it by ToM before and after treatment, the results showed that vmPFCtDCS can effectively improve the patients’ social ability and social cognitive function compared with the temporoparietal junction (TPJ) stimulation and false stimulation.

According to existing studies, TMS and tDCS have different mechanisms of action, but both have similar positive effects on patients with ASD. Sokhadze et al. (54) treated 112 high-functioning ASD whose IQ > 80 with low-frequency (1.0 Hz) rTMS and evaluated them with ABC and RBS-R before and after treatment. The motor accuracy and core symptoms such as stereotypical behavior and narrow interest of the experimental group were improved. Especially in the 18-week rTMS group, the patient’s cognitive abilities such as attention, discrimination, and executive function improved. Ni et al. (55) applied high-frequency iTBS (50 Hz) on bilateral DLPFC of patients with ASD, the results showed that patients’ anxiety, social disorder, stereotypical behavior, and cognitive function were improved. Of the 15 included studies on rTMS treatment, 9 of them (50–57, 59) selected the DLPFC as the target of stimulation, which appears to be the most favored site for rTMS treatment. This may be due to the fact that DLPFC mainly involves cognitive functions such as short-term memory, decision-making, and execution (66, 67). However, in children with ASD, due to the abnormal structure of the “microcolumn,” the inhibition of the cortex is weakened, and the excitability of the cortex is increased, which leads to the weakened connection between the anterior cingulate cortex and the DLPFC, and the brain’s processing ability to abnormal neural responses and wrong behaviors is decreased, and the brain is unable to make the real-time adjustment to the abnormal neural responses and wrong behaviors. Over time, it can lead to a decline in the executive ability of the child (68–70). By stimulating part of the brain and acting on the neural network around the “micro column,” TMS temporarily improves its inhibitory effect on the cortex, restores the connection between the anterior cingulate cortex and the DLPFC, reestablishes the monitoring-feedback system of the brain for abnormal behaviors and reactions, and improves the problem behaviors of patients. Moreover, TMS not only affects the brain regions stimulated directly, but also related brain regions, and strengthens the functional connections between these brain regions by virtue of the interconnected characteristics of the brain neural network (71). Currently, it is encouraging to see a diversity of therapeutic targets for TMS. Panerai et al. (39) applied high-frequency rTMS (8 Hz) to the left premotor cortex (PrMC) of patients with low-functioning ASD. The results were revised using the psychoeducational profile-revised (PEP-R) scale for children with ASD. The results showed that hand-eye coordination training was significantly improved when combined with high-frequency rTMS on the left PrMC. Peter et al. (19) used high-frequency rTMS (5 Hz) in the medial prefrontal cortex (mPFC) of high-functioning ASD. After 10 days of treatment, the patient’s social ability and communication skills were improved. Studies have shown that PrMC, especially left PrMC, is related to motor attention, tool use, hand-eye coordination, and other functions, mPFC is closely related to social and cognitive function (72–75).

It is worth noting that although most current studies use scale form to evaluate the therapeutic effect, the changes in scale data sometimes cannot objectively show the specific impact of NIBS on the brain function of patients with ASD. At present, it has become a novel measurement trend to observe the intervention effect of NIBS by biological means. Electrophysiological methods such as electroencephalography, event-related potential (ERP), and other measurements can objectively reflect the neuroregulatory effect of NIBS on patients with ASD, and more directly reflect the regulatory effect of NIBS on brain connectivity.

The above studies indicated that the application of tDCS in ASD intervention, patients with ASD who under 18 years of age were selected, and the majority were male. Choose more 1 mA or 1.5 mA anode stimulation to improve the core symptoms of ASD. However, 1.5 mA cathodic stimulation was used to improve the irritability and hyperactivity of patients with ASD, and 20 min was usually selected for each treatment. Selecting different cortical areas of the brain can also produce different therapeutic effects: applying to DLPFC can improve social impairment, abnormal behavior performance, short-term memory, etc. and applying to vmPFC can improve social impairment and cognitive level.

The patients selected for TMS were mostly male patients with high-functioning ASD, and most of them were adults or adolescents, which may be because TMS requires the cooperation of patients with higher cognitive function to complete the treatment, while adolescent or adult patients’ Neurodevelopmental function is relatively complete, so it is suitable for TMS. As far as application parameters are concerned, low frequency (1 Hz or 0.5 Hz) is used more to improve core symptoms of ASD. The treatment time is also closely related to the treatment effect. The treatment effect of 18 weeks is better than that of 6 weeks and 12 weeks, and the lasting effect is longer. High-frequency rTMS is mostly used to improve anxiety, promote social skills and communication skills, and improve the effectiveness of rehabilitation training, etc. Moreover, it is applied in different cortical areas of the brain, and different effects are obtained: application in DLPFC can improve stereotyped behavior, executive function, irritability, etc.; application in PrMC can improve language function and improve the effect of hand-eye coordination training; application in mPFC can improve social interaction ability, emotional state, etc.

## 5. Limitations

There are certain limitations in this study. The current clinical research on the treatment of ASD with NIBS is complex and diverse, and there are many difficulties, which makes the existing medical evidence difficult to meet the needs of reality in terms of quantity and quality, resulting in a greatly reduced accuracy of evaluation results. Meta-analysis is a retrospective observational study and cannot replace systematic, comprehensive, and in-depth clinical trials. In addition, high-strength medical evidence can improve the quality of meta-analysis. However, the literature included in this research has been published, and there is a lack of relevant gray literature, such as unpublished literature, special academic conference reports, etc. Among the 22 kinds of literature included, most of them were of low quality, and most of the studies did not adopt the double-blind method, thus reducing the strength of the conclusions of this study. Some of the included studies used a waiting group rather than a sham stimulus as a control, which may make the effect less objective. In addition, more than 80% of the studies were conducted by self-report or caregiver-report (mainly based on parent reports), therefore, results from behavioral measures may be limited by informant- vs. self-reporting. Close family members are often used as informants. However, many family members of individuals with ASD carry the diagnosis or demonstrate autistic traits without meeting the criteria of the disorder and often underreport symptoms in others that they experience. Additionally, due to interpersonal and social deficits observed in ASD, self-appraisal of social/emotional symptoms can be uniquely challenging.

Meta-analysis chooses scale scores such as ABC, ATEC, CARS, and RBS-R as evaluation indicators. Although they are simple and practical, they are greatly affected by the subjective factors of the evaluators, which may cause bias in the results and lead to unreliable conclusions. For example, the meta-analysis found that after NIBS treatment, there were no significant differences in CARS scores between the two groups. However, this result should be considered with caution due to the limited number of studies included, the small trial size, and inconsistent baseline patient characteristics. It is recommended that further large sample RCTS be performed to confirm the clinical efficacy of NIBS in patients with ASD, so as to better guide clinical decision-making.

## 6. Conclusion

Current studies have shown that rTMS and tDCS act on the same cerebral cortex region, producing highly overlapping therapeutic effects. It can be found from various studies that DLPFC stimulation can affect a wide range of areas and has certain effects on the core symptoms and cognitive functions of patients with ASD. Therefore, DLPFC is currently an important brain region where NIBS acts on patients with ASD. Encouragingly, stimulating other areas of the brain has also shown considerable therapeutic effects, but this needs to be seen in larger clinical studies. Meanwhile, the duration, intensity, interval, and other parameters of NIBS, as well as the accompanying treatment methods and individual characteristics, still need to be further discussed and studied. The dose, duration, and location of neuromodulation applied to different ASD children are different, and the research on the individual aspects of the treated children can become a new focus, especially in younger and lower-functioning patients with autism. In addition, it is strongly recommended that the medical history, current medication or psychotherapy, and risk–benefit ratio should be carefully assessed. Given that NIBS not only affects the stimulation site but also modulates other brain regions, future studies should carefully monitor the behavioral and physiological domains of patients in the long-term follow-up periods for any potential NIBS-induced negative effects. Although so far there are few reports on the side effects of NIBS, and the symptoms are relatively mild and easy to eliminate since most ASD patients affected by NIBS are children, long-term continuous observation and testing are needed in terms of safety and tolerance. In addition, some children with ASD will have epilepsy, attention deficit hyperactivity disorder, and other aspects of medication. How to achieve collaborative treatment under the condition of medication, or how to use NIBS treatment to relieve the side effects of drugs, to achieve the best therapeutic effect, will also become a new direction of research.

In summary, the existing results suggest that the therapeutic effect of NIBS on patients with ASD is limited, relevant clinical evidence is still insufficient and clear evidence of long-term efficacy is lacking. At this stage, NIBS cannot be recommended as a viable or evidence-based treatment for ASD. However, it is undeniable that the NIBS method is a promising treatment technique. In future clinical studies, it is necessary to conduct large-scale multi-center randomized double-blind controlled trials to establish safe and effective stimulation parameters, select homogenous research objects, and specify treatment plans for specific symptoms, in order to bring more powerful clinical evidence for patients with ASD.

## Author contributions

AL to conceived, designed, and wrote the articles. CG and BW carried out the literature retrieval and collection, literature content, data extraction, and quality evaluation. JS undertook data processing, applied RevMan software to draw relevant charts, and related statistics processing. ZJ was responsible for the quality control and proofreading of the articles. All authors contributed to the article and approved the submitted version.

## Funding

This study was funded by Excellent discipline team project of Jiamusi University (JDXKTD—2019006).

## Conflict of interest

The authors declare that the research was conducted in the absence of any commercial or financial relationships that could be construed as a potential conflict of interest.

## Publisher’s note

All claims expressed in this article are solely those of the authors and do not necessarily represent those of their affiliated organizations, or those of the publisher, the editors and the reviewers. Any product that may be evaluated in this article, or claim that may be made by its manufacturer, is not guaranteed or endorsed by the publisher.
